# Supplementary material for: Complexity of NAC Action as an Antidiabetic Agent: Opposing Effects of Oxidative and Reductive Stress on Insulin Secretion and Insulin Signaling
Source: Int J Mol Sci. 2022 Mar 9;23(6):2965. doi: 10.3390/ijms23062965 (PMC8950759; doi:10.3390/ijms23062965)
Supplement: Supplementary file 1 [file ijms-23-02965-s001.zip › ijms-1600168-supplementary.pdf]

Supplementary material

Table S1. Metabolic enzymes that were de-glutathionylated by NAC.

**Gene name**	**Protein name**	**Protein ID**
Asah1	Acid ceramidase	Q6P7S1
Aprt	Adenine phosphoribosyltransferase	P36972
Ahcy	Adenosylhomocysteinase	P10760
Aldh3a1	Aldehyde dehydrogenase	P11883
Hadha	Aldehyde dehydrogenase	Q64428
Akr1b1	Aldose reductase	P07943
Eno1	Alpha-enolase	P04764
Pfkm	ATP-dependent 6-phosphofructokinase, muscle type	P47858
Eno3	Beta-enolase	P15429
Phgdh	D-3-phosphoglycerate dehydrogenase	O08651
Fasn	Fatty acid synthase	P12785
Aldoa	Fructose-bisphosphate aldolase A	P05065
Glud1	Glutamate dehydrogenase 1, mitochondrial	P10860
Gapdh	Glyceraldehyde-3-phosphate dehydrogenase	P04797
Oat	Glyceraldehyde-3-phosphate dehydrogenase	P04182
Ldha	L-lactate dehydrogenase A chain	P04642
Mdh2	Malate dehydrogenase, mitochondrial	P04636
Cyb5r3	NADH-cytochrome b5 reductase 3	P20070
Hsd17b4	Peroxisomal multifunctional enzyme type 2	P97852
Pgk1	Phosphoglycerate kinase 1	P16617
Pgam1	Phosphoglycerate mutase 1	P25113
Pccb	Propionyl-CoA carboxylase beta chain, mitochondrial	P07633
Pkm	Pyruvate kinase PKM	P11980
Aldh9a1	Pyruvate kinase PKM	Q9JLJ3
Tkt	Transketolase	P50137
Atp5b	Transketolase	P10719
Hadhb	Trifunctional enzyme subunit beta, mitochondrial	Q60587
Tpi1	Triosephosphate isomerase	P48500
Ugdh	UDP-glucose 6-dehydrogenase	O70199

Table S2. Signaling proteins that were de-glutathionylated by NAC.

Gene name	Protein name	Protein ID
Ywhab	14-3-3 protein beta/alpha	P35213
Ywhae	14-3-3 protein epsilon	P62260
Ywhag	14-3-3 protein gamma	P61983
Ywhaz	14-3-3 protein zeta/delta	P63102
Anxa1	Annexin A1	P07150
Anxa2	Annexin A2	Q07936
Anxa5	Annexin A5	P14668
Anxa6	Annexin A6	P48037
Csnk1a1	Casein kinase I isoform alpha	P97633
Csnk1d	Casein kinase I isoform delta	Q06486
Cdc42	Cell division control protein 42 homolog	Q8CFN2
Gkap1	G kinase-anchoring protein 1	Q5XIG5
Sncg	Gamma-synuclein	Q63544
Gnb2	Guanine nucleotide-binding protein G(I)/G(S)/G(T) subunit beta-2	P54313
Lrrc59	Leucine-rich repeat-containing protein 59	Q5RJR8
Lifr	Leukemia inhibitory factor receptor	O70535
Mif	Macrophage migration inhibitory factor	P30904
Mapk1	Mitogen-activated protein kinase 1	P63086
Ppia	Peptidyl-prolyl cis-trans isomerase A	P10111
Fkbp1a	Peptidyl-prolyl cis-trans isomerase FKBP1A	Q62658
Pebp1	Phosphatidylethanolamine-binding protein 1	P31044
Pdcd6ip	Programmed cell death 6-interacting protein	Q9QZA2
Mtdh	Protein LYRIC	Q9Z1W6
Ndrg1	Protein NDRG1	Q6JE36
S100a10	Protein S100-A10	P05943
Rap1b;Rap1a	Ras-related protein Rap-1b	Q62636
Arhgdia	Rho GDP-dissociation inhibitor 1	Q5XI73
Rhoa	Transforming protein RhoA	P61589

Table S3. Trafficking proteins that were de-glutathionylated by NAC.

Gene name	Protein name	Protein ID
Arf1	ADP-ribosylation factor 1	P84079
Arf4	ADP-ribosylation factor 4	P61751
Cltc	Clathrin heavy chain 1	P11442
Copg1	Coatomer subunit gamma-1	Q4AEF8
Ehd2	EH domain-containing protein 2	Q4V8H8
Ran	GTP-binding nuclear protein Ran	P62828
Rab11a	Ras-related protein Rab-11A	P62494
Rab5a	Ras-related protein Rab-5A	M0RC99
Rab7a	Ras-related protein Rab-7a	P09527
Snx1	Sorting nexin-1	Q99N27
Snx24	Sorting nexin-24	Q5U2S5
Vcp	Transitional endoplasmic reticulum ATPase	P46462

Table S4. Proteins involved in: Folding, repair and unfolded-protein response, that were de-glutathionylated by NAC.

Gene name	Protein name	Protein ID
Hspe1	10 kDa heat shock protein, mitochondrial	P26772
Hspd1	60 kDa heat shock protein, mitochondrial	P63039
Hspa5	78 kDa glucose-regulated protein	P06761
Calr	Calreticulin	P18418
Dnajb11	DnaJ homolog subfamily B member 11	Q6TUG0
Hsp90b1	Endoplasmin	Q66HD0
Hspa8	Heat shock cognate 71 kDa protein	P63018
Hspb1	Heat shock protein beta-1	P42930
Hsp90aa1	Heat shock protein HSP 90-alpha	P82995
Hsp90ab1	Heat shock protein HSP 90-beta	P34058
St13	Hsc70-interacting protein	P50503
Ppib	Peptidyl-prolyl cis-trans isomerase B	P24368
P4hb	Protein disulfide-isomerase	P04785
Pcmt1	Protein-L-isoaspartate(D-aspartate) O-methyltransferase	P22062
Serpinh1	Serpin H1	P29457
Hspa9	Stress-70 protein, mitochondrial	P48721
Stip1	Stress-induced-phosphoprotein 1	O35814
Tcp1	T-complex protein 1 subunit alpha	P28480
Cct2	T-complex protein 1 subunit beta	Q5XIM9
Cct4	T-complex protein 1 subunit delta	Q7TPB1
